# Inferring Novel Autophagy Regulators Based on Transcription Factors and Non-Coding RNAs Coordinated Regulatory Network

**DOI:** 10.3390/cells7110194

**Published:** 2018-11-02

**Authors:** Shuyuan Wang, Wencan Wang, Qianqian Meng, Shunheng Zhou, Haizhou Liu, Xueyan Ma, Xu Zhou, Hui Liu, Xiaowen Chen, Wei Jiang

**Affiliations:** 1 College of Bioinformatics Science and Technology, Harbin Medical University, Harbin 150081, China; bioccwsy@163.com (S.W.); wangwencan1314@163.com (W.W.); mqq1992hmu@163.com (Q.M.); 18345550297@163.com (X.M.); biomathzx@163.com (X.Z.); liuhui870320@163.com (H.L.); 2 College of Automation Engineering, Nanjing University of Aeronautics and Astronautics, Nanjing 211106, China; zhoushunheng@163.com (S.Z.); liuhaizhou2015@126.com (H.L.)

**Keywords:** autophagy regulator, transcriptional factor, non-coding RNA, regulatory network, RWR algorithm

## Abstract

Autophagy is a complex cellular digestion process involving multiple regulators. Compared to post-translational autophagy regulators, limited information is now available about transcriptional and post-transcriptional regulators such as transcription factors (TFs) and non-coding RNAs (ncRNAs). In this study, we proposed a computational method to infer novel autophagy-associated TFs, micro RNAs (miRNAs) and long non-coding RNAs (lncRNAs) based on TFs and ncRNAs coordinated regulatory (TNCR) network. First, we constructed a comprehensive TNCR network, including 155 TFs, 681 miRNAs and 1332 lncRNAs. Next, we gathered the known autophagy-associated factors, including TFs, miRNAs and lncRNAs, from public data resources. Then, the random walk with restart (RWR) algorithm was conducted on the TNCR network by using the known autophagy-associated factors as seeds and novel autophagy regulators were finally prioritized. Leave-one-out cross-validation (LOOCV) produced an area under the curve (AUC) of 0.889. In addition, functional analysis of the top 100 ranked regulators, including 55 TFs, 26 miRNAs and 19 lncRNAs, demonstrated that these regulators were significantly enriched in cell death related functions and had significant semantic similarity with autophagy-related Gene Ontology (GO) terms. Finally, extensive literature surveys demonstrated the credibility of the predicted autophagy regulators. In total, we presented a computational method to infer credible autophagy regulators of transcriptional factors and non-coding RNAs, which would improve the understanding of processes of autophagy and cell death and provide potential pharmacological targets to autophagy-related diseases.

## 1. Introduction

Autophagy is a process of cytoplasmic degradation that is essential in homeostasis and stress-response, as well as in protein degradation and organelles turnover [1]. The regulation of autophagy is critical in human health and disease. Both its insufficient and overdriven activity can disturb the body functions, including causing cancers. For example, autophagy deficiency causes oxidative stress and genome instability which is a known cause of cancer initiation and progression [2] and up-regulation of autophagy in RAS-transformed cancer cells promotes their growth, survival, tumorigenesis invasion, and metastasis [3]. The process of autophagy involves multiple kinds of regulators, including autophagy-related (*ATG*) genes, ATG proteins and non-coding RNAs (ncRNAs). For instance, the autophagy database archived a list of 582 experimentally demonstrated ATG proteins [4] and Wu et al. provided a comprehensive bioinformatics resource to dissect ncRNA-mediated autophagy interactions [5]. In addition, regulation of autophagy by targeting autophagy regulators is a promising strategy for cancer therapy [6]. For example, temsirolimus could significantly prolong progression-free survival of mantle cell lymphoma (MCL) patients by inhibiting the mechanistic target of rapamycin (mTOR) protein, a post-translational autophagy regulator [7].

Currently, post-translational autophagy regulators, such as ATG proteins, are well known while limited information is available about transcriptional and post-transcriptional regulators, such as transcription factors (TFs) and ncRNAs [8]. Inferring that novel transcriptional and post-transcriptional autophagy regulators will help to dissect the autophagy regulation mechanisms and provide possible pharmacological targets to regulate autophagy. The TFs and ncRNAs coordinated regulatory (TNCR) network has demonstrated its power as a tool to study biological issues such as regulatory pathways in human diseases, classifiers for drug resistance and so on [9,10,11]. For example, Liang et al. performed deconvolution on the transcriptional network and demonstrated that BACH1 was the master regulator of breast cancer bone metastasis [12]. Wang et al. identified disease-related regulatory cascades by dissecting the TF and miRNA regulatory network, which helped understand the pathogenesis [13]. Recently, lncRNAs were found to be targeted by miRNAs and functioned as miRNA sponges to attenuate the inhibition ability of miRNAs to mRNAs. Furthermore, lncRNAs were also shown to play crucial roles in the regulation of gene expression at transcriptional and post-transcriptional levels [14,15]. Thus, lncRNAs introduce an extra layer of complexity to the TNCR network, enhancing the analytical ability of the regulatory network.

In this study, we proposed a computational method to predict novel autophagy-associated TFs, miRNAs and lncRNAs based on the TNCR network. First, experimentally verified transcriptional and post-transcriptional regulatory relationships among TFs, miRNAs and lncRNAs were collected and a comprehensive regulatory network was constructed. Next, the known autophagy-associated TFs, miRNAs and lncRNAs were gathered from public data resources. The random walk with restart (RWR) algorithm was implemented on the regulatory network to prioritize autophagy regulators. Leave-one-out cross-validation (LOOCV) achieved an area under the curve (AUC) of 0.889. Functional enrichment analyses and extensive literature surveys demonstrated the credibility of predicted regulators. Altogether, we presented a computational method of inferring credible autophagy regulators and we believed that this would help improve the understanding of the autophagy regulation mechanisms.

## 2. Materials and Methods

### 2.1. Construction of a Comprehensive TNCR Network

We integrated five types of experimentally verified transcriptional and post-transcriptional regulatory relationships among TFs, miRNAs and lncRNAs, including TF-miRNA, TF-lncRNA, miRNA-lncRNA, miRNA-TF, lncRNA-TF. The TFs regulations of miRNAs were downloaded from the database TransmiR, which manually surveyed literature and recorded experimentally supported TF-miRNA regulation [16]. The TFs regulations of lncRNAs were obtained from the database ChIPBase, which decoded the transcriptional regulation of lncRNAs from ChIP-seq data in diverse tissues and cell lines [17]. Here, only TF-lncRNA regulations that were identified in more than 20 datasets were retained. In order to improve the credibility of the regulations, we also used the TRANSFAC Match program to assure transcription factor binding sites (TFBS) in lncRNA sequences [18] using minimum false-positive profiles of vertebrate high quality matrices. The final TF-lncRNA regulations were obtained by intersecting the ChIPBase data source with the TRANSFAC results. The miRNAs regulations of TFs were integrated from two databases, miRecords [19] and miRTarBase [20]. Both of these two databases collected experimentally validated miRNA-target interactions, and we retained the union set of the relationships presented in these two databases. The miRNAs regulations of lncRNAs were derived from LncBase v2 which provided experimentally supported and in silico predicted miRNA recognition elements (MREs) on lncRNAs [21]. We retained the interactions presented in the experimental module and the prediction scores should have been equal to or greater than 0.95. The lncRNAs regulations of TFs were downloaded from LncReg [22] and LncRNA2Target [23]. The database LncReg collected validated lncRNA-associated regulatory entries while LncRNA2Target curated differentially expressed genes after the lncRNA knockdown or overexpression. We kept the union set of the lncRNAs regulations of TFs which were provided by these two databases. Integrating all of the above regulations, we constructed a comprehensive TNCR network.

### 2.2. Collection of Known Autophagy Regulators

The known autophagy-associated TFs, miRNAs and lncRNAs were collected from public data resources. We first obtained human genes in autophagy related Gene Ontology (GO) terms from the AmiGO-2 database. Next, we downloaded the human autophagy-associated genes from the autophagy database [4], a multifaceted online resource providing information on genes and proteins related to autophagy across several eukaryotic species. The union set of these two gene sets were regarded as known autophagy-associated genes. As for autophagy-associated miRNAs and lncRNAs, we resorted to the database ncRDeathDB, a comprehensive bioinformatics resource archiving ncRNA-associated cell death interactions and picked up the autophagy-associated miRNAs and lncRNAs [5]. All the autophagy-associated genes, miRNAs and lncRNAs we obtained were mapped onto the TNCR network, and the intersections were regarded as seeds for RWR algorithm.

### 2.3. Prioritization of Novel Autophagy Regulators with the RWR Method

We performed the RWR method on the constructed TNCR network to prioritize novel autophagy regulators. The RWR method simulates a random walker that starts on given seed nodes and transits randomly from the current node to neighboring nodes in the network with the restart probability to teleport to the start nodes. Here, the known autophagy regulators were used as seed nodes. We denoted *P*_0_ as the initial probability vector and *P_t_* as a vector in which the *i*-th element held the probability of finding the random walker at node *i* in step *t*. Let *α* be the restart probability of the random walk in each step at the source nodes. *W* denotes the probability transition matrix and is derived from the adjacency matrix of the TNCR network. The formula is defined as:

(1) $$\;w\left(i,j\right)=\{\begin{array}{cc} A\left(i,j\right)/\underset{j}{\sum }A\left(i,j\right), & if\;\underset{j}{\sum }A\left(i,j\right)\neq 0\\ 0, & otherwise \end{array}\;$$

where *w* (*i*, *j*) represents the element in the probability transition matrix, and *A* (*i*, *j*) represents the element in the adjacency matrix. The probability vector in step *t* + 1 can be described as follows:

(2) $$\;p_{t+1}=\left(1-\alpha \right)wp_{t+1}+\alpha p_{0}\;$$

Based upon the previous work, the restart probability (*α*) was set as 0.5, and the initial probability (*P*_0_) of each seed node was set as 1/*n* (where *n* is the number of seed autophagy regulators) while the initial probability of all non-seed nodes was set as zero [24,25]. With the iteration steps going on, the probability of the RWR algorithm will become stable. We defined the stable probability as $P_{\infty }$ when the difference between *P_t_* and *P_t+1_* was less than 10^−10^. The stable probability of $P_{\infty }$ can be used as a measure of proximity to the seed regulators. If $P_{\infty }\left(node_{i}\right)>P_{\infty }\left(node_{j}\right)$, then node*_i_* will be in closer proximity to the seed regulators in the regulatory network than node*_j_*. As a result, all candidate nodes in the regulatory network can be ranked according to $P_{\infty }$ and the top ranked elements can be expected to have a high probability of being associated with autophagy.

### 2.4. Functional Analysis for Predicted Autophagy Regulators

To demonstrate the credibility of the proposed prediction method, we performed functional analysis for the predicted autophagy regulators. We first retrieved the top 100 ranked regulator candidates (excluding seeds), including TFs, miRNAs and lncRNAs, and performed separately the functional enrichment analyses. For the obtained TFs, we used DAVID to perform GO and Kyoto Encyclopedia of Genes and Genomes (KEGG) pathway enrichment analysis [26]. For the obtained miRNAs, we collected the experimentally verified miRNA targets from the miRecords [19] and miRTarBase [20]; we then used the union set of the miRNA targets to perform GO and KEGG pathway enrichment analysis with DAVID. For the obtained lncRNAs, we utilized the recently developed function annotation tool of non-coding RNA (FARNA), a knowledgebase of inferred functions of human ncRNA transcripts, to implement function annotation analysis. We searched the FARNA database by using each obtained lncRNA, and retrieved promoter-associated transcription factors and transcription co-factors for the lncRNA. Then, all the obtained transcription factors and transcription co-factors were inputted into DAVID to perform GO and KEGG pathway enrichment analysis. In addition, we also performed GO enrichment analysis for the known autophagy-associated TFs, miRNAs and lncRNAs separately, as described above. The union set of the significant GO categories were considered as the autophagy related GO terms. All these DAVID analyses adopted the same criteria that the biological process (BP) category was used for GO analysis, and the significance of enrichment was set at *p*-value < 0.05. Finally, we calculated the functional similarity scores between the GO terms enriched in the predicted autophagy regulators and the autophagy related GO terms. The computational procedure was implemented using R package GOSemSim [27] and the rcmax method was chosen as a combined method for aggregating multiple GO terms. We also performed 1000 random tests to evaluate the significance of obtained functional similarity scores. In each random test, we randomly chose the same number of GO terms as in the real situation and calculated the functional similarity scores as above. The statistical *p*-value was calculated as the ratio of random functional similarity scores higher than the real functional similarity score.

## 3. Results

### 3.1. Characteristics of the TNCR Network

**Figure 1:**
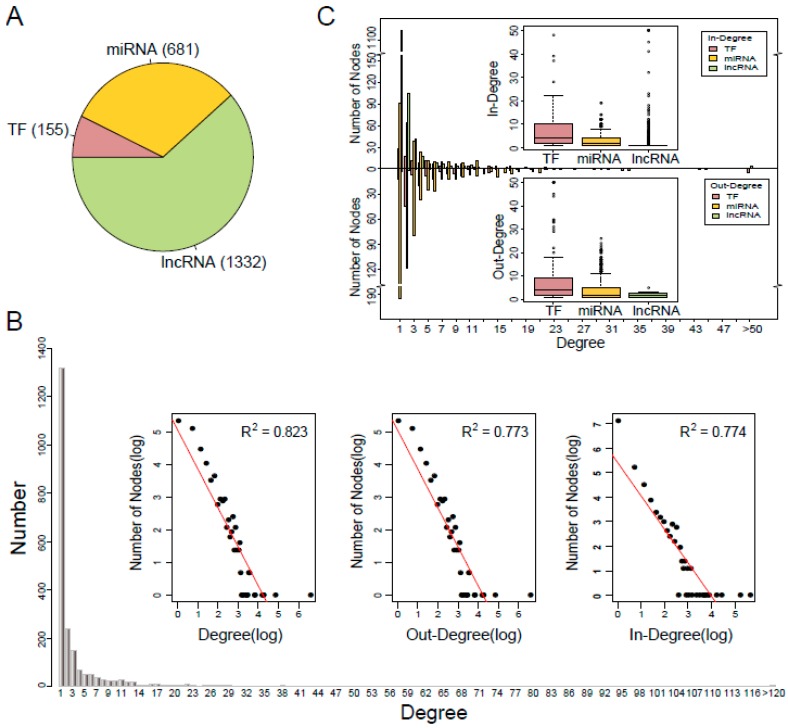
Characteristics of the TFs and ncRNAs coordinated regulatory (TNCR) network. (**A**) Proportion of transcription factor (TF), microRNA (miRNA) and long non-coding RNA (lncRNA) in the TNCR network. (**B**) Degree distribution of all nodes in the TNCR network and the log-log plots for the degree, out-degree and in-degree distributions of all nodes. (**C**) In-degree and out-degree distributions of TFs, miRNAs and lncRNAs in the TNCR network.

In this study, we integrated five types of experimentally verified transcriptional and post-transcriptional regulatory relationships from public data resources and constructed a comprehensive TNCR network (see Materials and Methods for details). The TNCR network comprised of 4529 edges, including 155 TFs, 681 miRNAs and 1332 lncRNAs (Figure 1A, Supplementary Table S1). To get an overview of the TNCR network, we examined the degree distribution of the network. As shown in Figure 1B, most nodes (50.4%) had degree one and few nodes had a high degree. In addition, the power-law distribution of the forms $y=327.4\times 10^{-1.31}\;\left(R^{2}=0.823\right)$, $y=157.4\times 10^{-1.19}\;\left(R^{2}=0.773\right)$ and $y=224.4\times 10^{-1.36}\;\left(R^{2}=0.774\right)$ were fitted for degree, out-degree and in-degree respectively. These results indicated that the TNCR network satisfied approximate scale-free topology which is the common feature of most biological networks [28]. Next, we further investigated the in-degree and out-degree distributions for TFs, miRNAs and lncRNAs, respectively (Figure 1C). In general, few nodes had very high degrees and many had low degrees, regardless of TFs, miRNAs or lncRNAs in-degree and out-degree. Furthermore, TFs had a higher median in-degree and out-degree than miRNAs and lncRNAs, which meant that TFs more likely acted as hubs in the TNCR network.

### 3.2. Performance Evaluation of the Proposed Method

By integrating data from AmiGO-2, the autophagy database and the ncRDeathDB, we obtained 1222 known autophagy regulators in total (Supplementary Table S2). After mapping these regulators onto the TNCR network, we finally got 178 autophagy regulators as seeds, including 25 TFs, 152 miRNAs and 1 lncRNAs (Supplementary Table S3). By performing the RWR method on the TNCR network with the seeds, we finally prioritized novel autophagy regulators.

**Figure 2:**
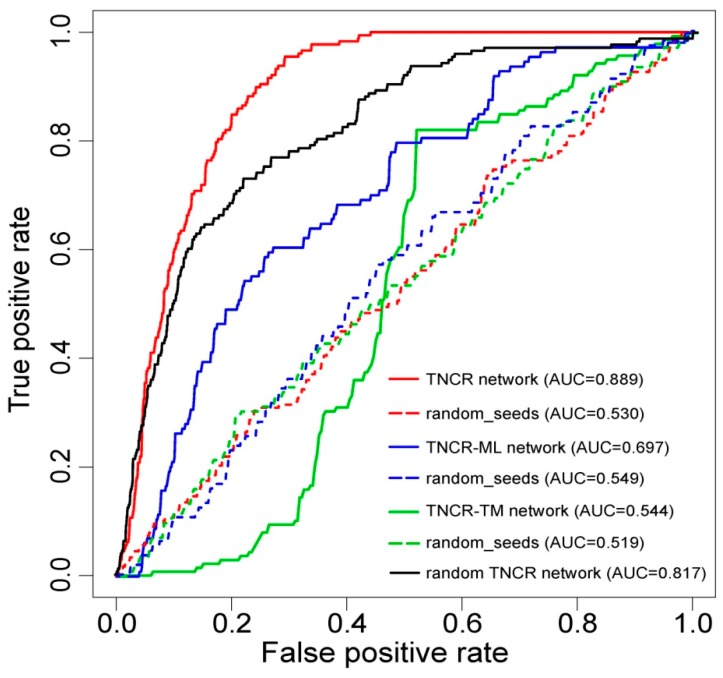
Receiver operating characteristic (ROC) curves and area under the curve (AUC) values for the random walk with restart (RWR) method on the whole, partial and random TNCR networks with real seeds and random seeds. The ROC curves were plotted and AUC values were calculated separately by leave-one-out cross-validation (LOOCV) for the TNCR network, TNCR-ML (miRNAs and lncRNAs only) network, TNCR-TM (TFs and miRNAs only) network and the random TNCR network with real and random seeds.

In order to evaluate the performance of our method for inferring autophagy regulators, we performed LOOCV analysis. Each known autophagy regulator was left out in turn as the test case and the other known autophagy regulators were taken as seeds. All the other nodes in the TNCR network were regarded as candidate autophagy regulators. Sensitivity and specificity were calculated for each threshold. Finally, a receiver operating characteristic (ROC) curve was plotted by varying the threshold and then the value of the AUC was calculated. Our method, tested on already known autophagy regulators, achieved an AUC of 0.889 (Figure 2), exhibiting excellent performance. Here, the TNCR network incorporated three kinds of regulators (TFs, miRNAs and lncRNAs) and five kinds of regulations (TF-miRNA, TF-lncRNA, miRNA-lncRNA, miRNA-TF and lncRNA-TF). To demonstrate the effectivity and reliability of the TNCR network, we compared the performance of partial TNCR networks. The AUCs were calculated for a TNCR-ML network (miRNAs and lncRNAs only) and a TNCR-TM network (TFs and miRNAs only) separately by performing LOOCV (the TNCR-TL network (TFs and lncRNAs only) was not analyzed because of missing seed regulators). The AUCs were 0.697 and 0.544 respectively, which were lower than those using the TNCR network (Figure 2). To further determine whether the results of the cross validation might have been generated by chance, we performed randomization tests. The seeds were generated randomly from candidate nodes in all three networks and the AUC values were calculated by performing LOOCV, as above. The AUC values under randomized tests were much lower than those in real situations (0.530, 0.549 and 0.519, respectively, for these three conditions), confirming the valid and reliable performance of autophagy regulator seeds in our method (Figure 2). We also performed RWR on 1000 degree-preserving randomized TNCR networks and the average value of the AUCs was calculated. As shown in Figure 2, the result based on the real TNCR network and the real seed nodes performed best.

The prioritization of all candidate autophagy regulators is provided in Supplementary Table S4. The top 100 ranked candidate regulators, including 55 TFs, 19 miRNAs and 26 lncRNAs, were further validated by literature mining, in which 52 regulators had been verified to be associated with autophagy in published papers (Supplementary Table S5). For example, the fifth ranked regulator MYC was recently proved to mitigate its oncogenic activity by chaperone-mediated autophagy (CMA) regulation [29] and the ninth ranked regulator XIST was determined to increase autophagy activity in non-small-cell lung cancer by regulation of ATG7 [30]. The extensive literature surveys demonstrated the feasibility of our method to predict autophagy regulators.

### 3.3. Functional Characteristics of Predicted Autophagy Regulators

**Figure 3:**
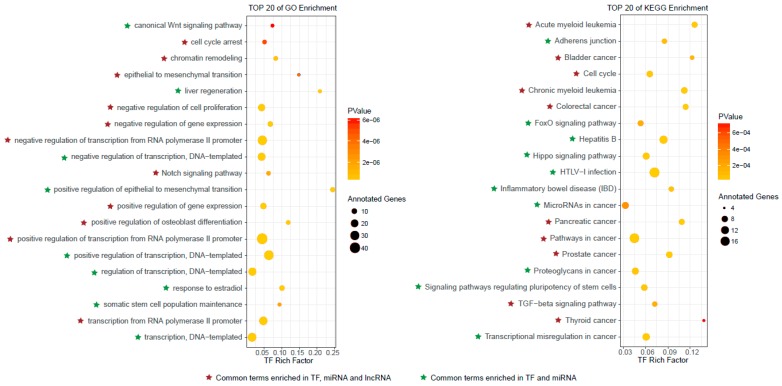
The top 20 Gene Ontology (GO) enrichment and Kyoto Encyclopedia of Genes and Genomes (KEGG) enrichment results for top ranked TFs. The common enriched GO terms and KEGG pathways among top ranked TFs, miRNAs and lncRNAs are marked.

**Figure 4:**
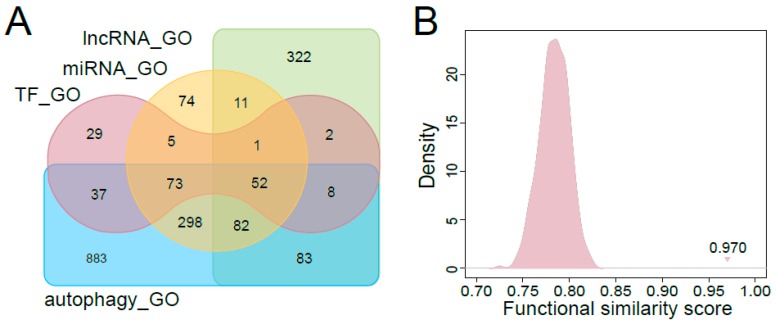
Evaluation of the top ranked regulators associated with autophagy. (**A**) Venn plot for the GO functional annotation comparison among the top ranked TFs, miRNAs, lncRNAs and the known autophagy-associated factors. (**B**) Distribution of random functional similarity scores for the top ranked TFs and the autophagy-associated factors. The triangle indicates the true functional similarity score for top ranked TFs and the known autophagy-associated factors.

The top 100 ranked candidate autophagy regulators were retrieved, including 55 TFs, 19 miRNAs and 26 lncRNAs (Supplementary Table S4), then the functional analyses were performed separately for these predicted autophagy regulators (see Materials and Methods for details). The top 20 significantly enriched GO terms and KEGG pathways for TFs are shown in Figure 3. We observed that some cell death related GO terms, such as cell cycle arrest and negative regulation of cell proliferation, were enriched by these top ranked TFs. Several significantly enriched KEGG pathways were also related to cell death, for instance, cell cycle and adherens junction. In addition, some cancer related pathways, such as colorectal cancer, prostate cancer and thyroid cancer, were also enriched, indicating that the autophagy regulators played important roles in cancer. This was consistent with previous studies [11,31,32]. The top 20 significantly enriched GO terms and KEGG pathways by the top ranked miRNAs and lncRNAs are shown in Figure S1 and Figure S2. Similar to the top ranked TFs, the cell death related GO terms and KEGG pathways, such as apoptotic process and cell proliferation, were also enriched by top ranked miRNAs and lncRNAs. Cancer related pathways, such as pancreatic cancer and small cell lung cancer, were enriched by top ranked miRNAs and lncRNAs. We observed that there were obvious overlaps among GO terms and KEGG pathways enriched by top ranked TFs, miRNAs and lncRNAs (Figure 3 and Figure 4A). All of the significantly enriched GO terms and KEGG pathways (*p*-value < 0.05) for top ranked TFs, miRNAs and lncRNAs were shown in Supplemental Table S6.

To further evaluate the top ranked regulators associated with autophagy, we compared the GO terms enriched by the top 100 ranked regulators with those enriched by known autophagy-associated factors (including protein-coding genes, miRNAs and lncRNAs). As shown in Figure 4A, the numbers of overlapping enriched GO terms among top-ranked TFs, miRNAs, lncRNAs and known autophagy-associated factors were high (the significantly enriched GO terms for known autophagy-associated factors were shown in Supplemental Table S7). We calculated the functional similarity scores between the GO terms enriched by the top 100 ranked regulators and the autophagy related GO terms. The functional similarity scores between the autophagy related GO terms and those enriched by top ranked TFs, miRNAs, lncRNAs were 0.970, 0.978 and 0.949, respectively. The random functional similarity scores for each kind of regulators, which were calculated by randomly choosing the same number of GO terms as in the real situation, were significantly lower than the real scores (Figure 4B, Figure S3). All these *p*-values were less than 2.2 × 10^−16^ (see Materials and Methods for details). This meant that the top ranked regulators were significantly associated with autophagy. The functional characteristics of the top ranked regulators indicated that our method was capable of identifying novel autophagy regulators.

## 4. Discussion

Autophagy is an intracellular catabolic process for maintaining homeostasis and involved systematic regulation at post-translational, transcriptional, and post-transcriptional levels [33]. Both its insufficient and overdriven functions can disturb intracellular homeostasis [34]. Thus, the regulation of autophagy is critical for body cells normal function. Although the knowledge of autophagy regulation is making certain progress, the landscape of autophagy regulators is far from completeness. In addition, autophagy demonstrates a promising therapeutic target in several pathologies [35]. Thus, identification of novel autophagy regulators is beneficial to targeted therapy of complex human diseases. Regulatory networks provide global views of the transmission of genetic information, and are proved to be powerful tools for studying biological issues. In this study, we conducted a computational method to infer novel autophagy regulators based on the regulatory network. We first constructed a comprehensive regulatory TNCR network that incorporated transcriptional and post-transcriptional regulators, including TFs, miRNAs and lncRNAs. Network topological analysis revealed that the degree distribution of the TNCR network approximately followed the power-law distribution. Then, the candidate autophagy regulators were ranked by implementing the RWR method on the TNCR network using the known autophagy regulators as seed nodes. The AUC values determined by LOOCV achieved 0.889, demonstrating the high credibility of our method for recovering known autophagy regulators. Furthermore, functional enrichment analyses revealed that the predicted autophagy regulators were associated with cell death related functional categories such as negative regulation of cell proliferation, cell death and cell cycle arrest. Significantly high functional semantic similarity scores were obtained between the obtained GO terms and the autophagy related GO terms. In addition, extensive literature surveys demonstrated that the top ranked regulators were verified to have associations with autophagy. All these results indicate that our approach is effective in inferring transcriptional and post-transcriptional autophagy regulators and that it would help to improve the understanding of the autophagy regulation mechanisms.

In the past several years, the landscape of TNCR networks has been described elaborately [12,13]. Several experimentally verified transcriptional and post-transcriptional regulatory databases have been developed, such as TransmiR [16], ChIPBase [17], miRTarBase [20] and so on. However, the exhaustive transcriptional and post-transcriptional regulatory relationships still need further elucidation. For example, the characterization of lncRNAs regulation of TFs is still at a primary level [36]. Furthermore, the competing endogenous RNA (ceRNA) relationships involved in TFs, miRNAs and lncRNAs provide further complex regulations among transcriptional and post-transcriptional factors which should be considered in the future analysis of TNCR network [37]. Our approach in this study was based on the general regulatory network TNCR; however, the autophagy plays tissue-specific and double-edged roles in the cellular homeostasis and survival. We believe that the performance of our approach would be improved if we use the data of a specific cancer. In addition, the comprehensiveness of seeds is critical for the performance of the RWR algorithm [38]. Currently, protein-coding regulators of the autophagic machinery are relatively well known, while few studies have been conducted on the non-coding RNA regulators, especially lncRNAs. With the abundance of research of autophagy related regulators, we will obtain comprehensive seed autophagy regulators, and provide more credible, verifiable autophagy regulators.

## Supplementary Materials

The following are available online at http://www.mdpi.com/2073-4409/7/11/194/s1.
