# Supplementary material for: Inferring Novel Autophagy Regulators Based on Transcription Factors and Non-Coding RNAs Coordinated Regulatory Network
Source: Cells. 2018 Nov 2;7(11):194. doi: 10.3390/cells7110194 (PMC6262548; doi:10.3390/cells7110194)
Supplement: Supplementary file 1 [file cells-07-00194-s001.zip › Supplementary Files/figure_S2.pdf]

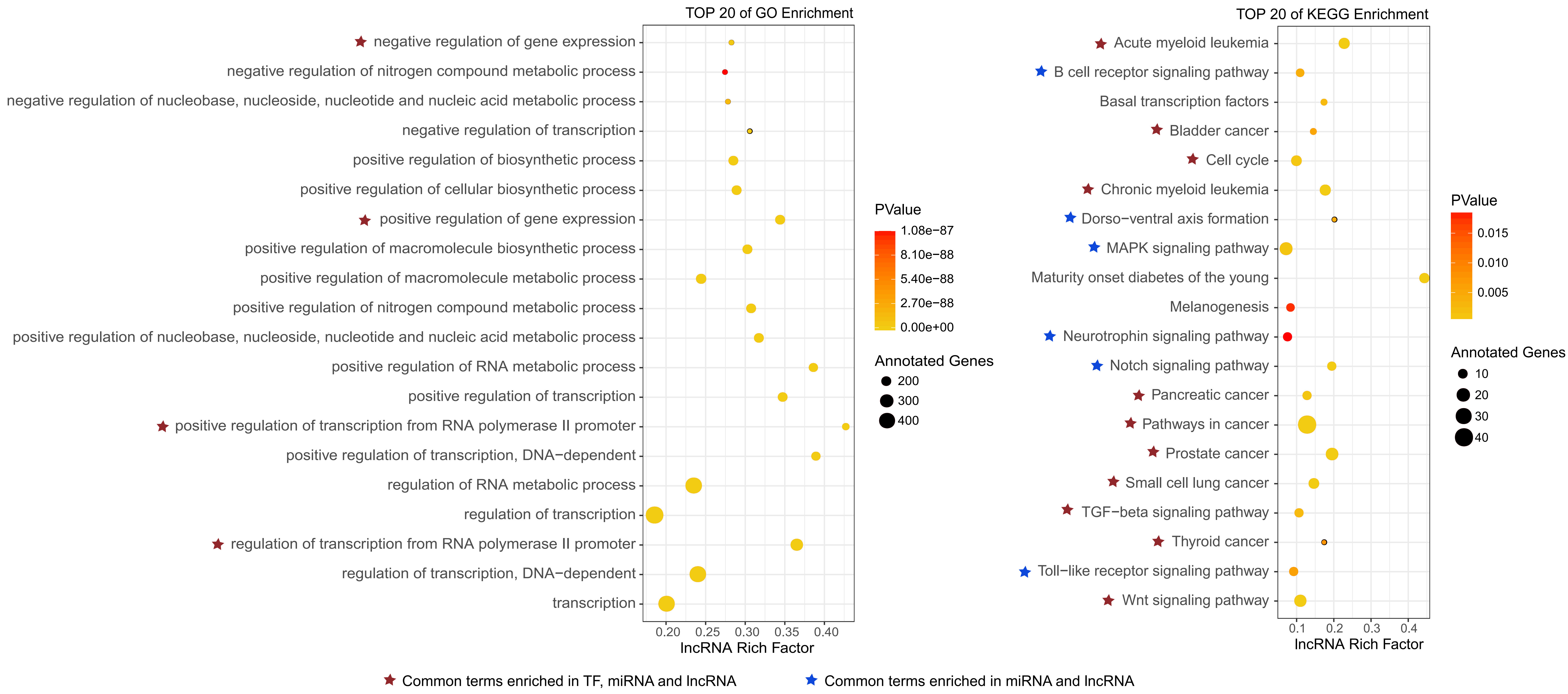
TOP 20 of GO Enrichment
negative regulation of gene expression
negative regulation of nitrogen compound metabolic process
negative regulation of nucleobase, nucleoside, nucleotide and nucleic acid metabolic process
negative regulation of transcription
positive regulation of biosynthetic process
positive regulation of cellular biosynthetic process
positive regulation of gene expression
positive regulation of macromolecule biosynthetic process
positive regulation of macromolecule metabolic process
positive regulation of nitrogen compound metabolic process
positive regulation of nucleobase, nucleoside, nucleotide and nucleic acid metabolic process
positive regulation of RNA metabolic process
positive regulation of transcription
positive regulation of transcription from RNA polymerase II promoter
positive regulation of transcription, DNA−dependent
regulation of RNA metabolic process
regulation of transcription
regulation of transcription from RNA polymerase II promoter
regulation of transcription, DNA−dependent
transcription
0.20 0.25 0.30 0.35 0.40
lncRNA Rich Factor
PValue
1.08e−87
8.10e−88
5.40e−88
2.70e−88
0.00e+00
Annotated Genes
200
300
400
TOP 20 of KEGG Enrichment
Acute myeloid leukemia
B cell receptor signaling pathway
Basal transcription factors
Bladder cancer
Cell cycle
Chronic myeloid leukemia
Dorso−ventral axis formation
MAPK signaling pathway
Maturity onset diabetes of the young
Melanogenesis
Neurotrophin signaling pathway
Notch signaling pathway
Pancreatic cancer
Pathways in cancer
Prostate cancer
Small cell lung cancer
TGF−beta signaling pathway
Thyroid cancer
Toll−like receptor signaling pathway
Wnt signaling pathway
0.1 0.2 0.3 0.4
lncRNA Rich Factor
PValue
0.015
0.010
0.005
Annotated Genes
10
20
30
40
Common terms enriched in TF, miRNA and lncRNA
Common terms enriched in miRNA and lncRNA
