# Supplementary figures and images for: Inferring Novel Autophagy Regulators Based on Transcription Factors and Non-Coding RNAs Coordinated Regulatory Network
Source: Cells. 2018 Nov 2;7(11):194. doi: 10.3390/cells7110194 (PMC6262548; doi:10.3390/cells7110194)

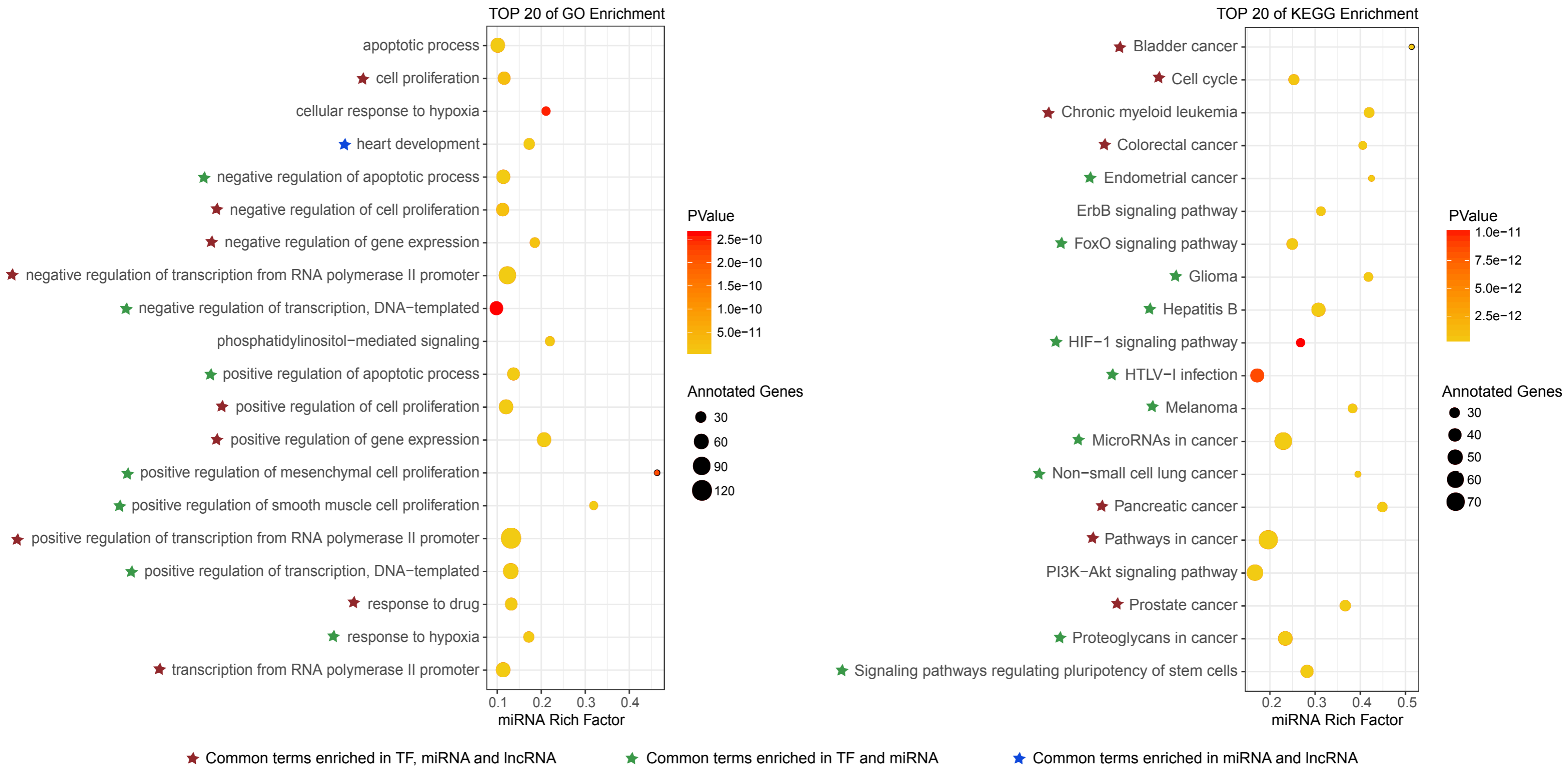

Supplement: Supplementary file 1 [file cells-07-00194-s001.zip › Supplementary Files/figure_S1.pdf]

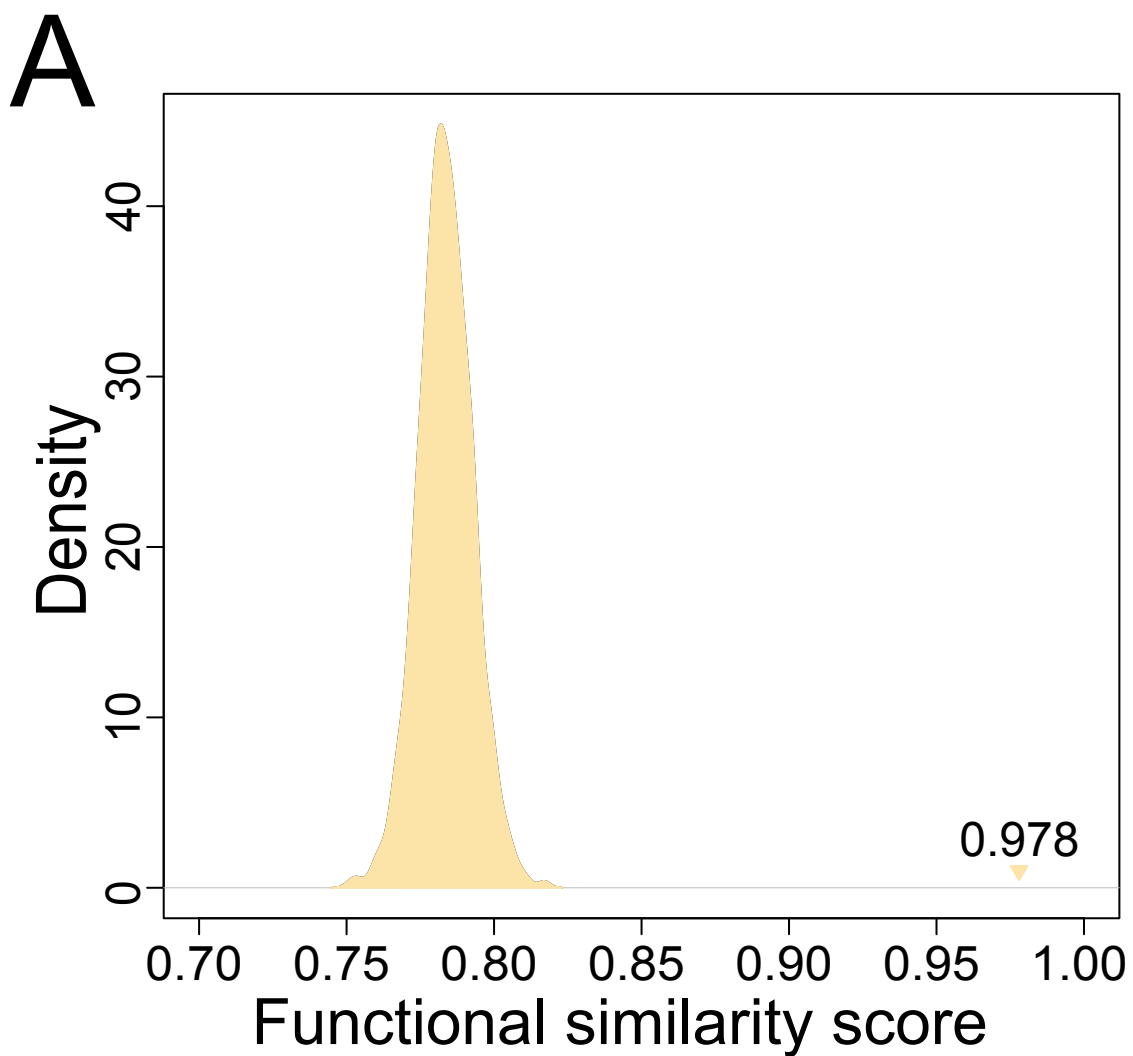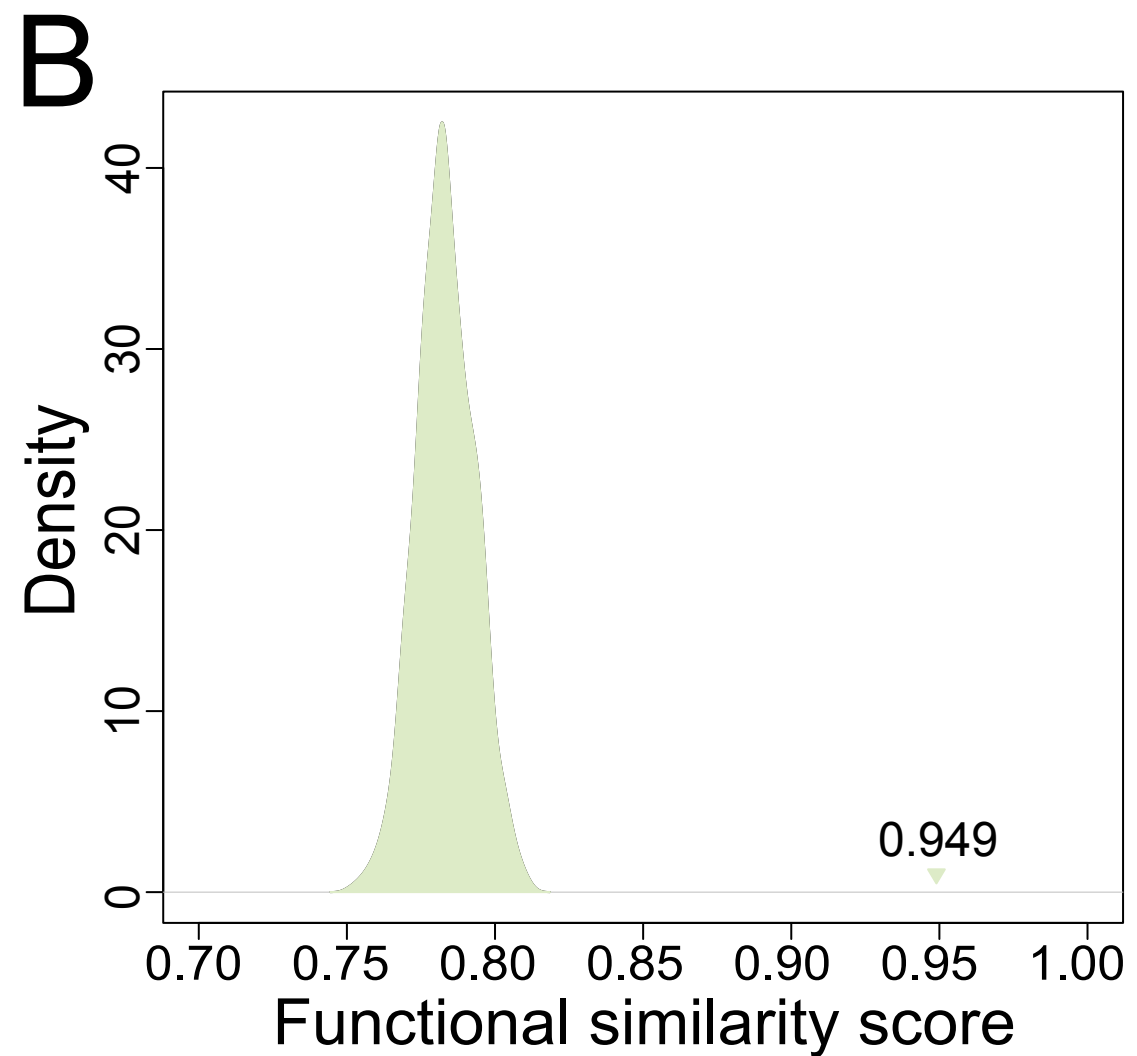

Supplement: Supplementary file 1 [file cells-07-00194-s001.zip › Supplementary Files/figure_S3.pdf]
